# Supplementary material for: Causal Relationship between Aging and Anorexia Nervosa: A White-Matter-Microstructure-Mediated Mendelian Randomization Analysis
Source: Biomedicines. 2024 Aug 16;12(8):1874. doi: 10.3390/biomedicines12081874 (PMC11351342; doi:10.3390/biomedicines12081874)
Supplement: Supplementary file 1 [file biomedicines-12-01874-s001.zip › Supplementary Figure/Figure S4/Singe SNP plot of MR analysis for TL to ubm-b-1937.pdf]

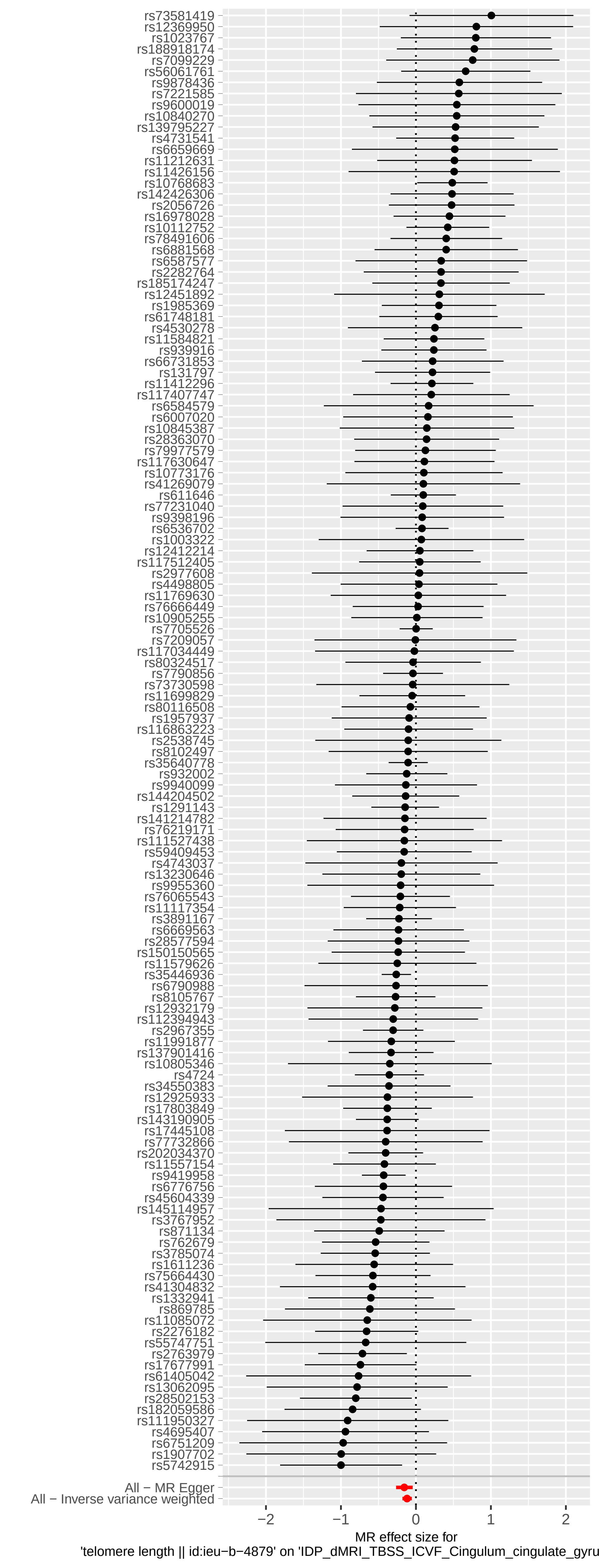
rs73581419
rs12369950
rs1023767
rs188918174
rs7099229
rs56061761
rs9878436
rs7221585
rs9600019
rs10840270
rs139795227
rs4731541
rs6659669
rs11212631
rs11426156
rs10768683
rs142426306
rs2056726
rs16978028
rs10112752
rs78491606
rs6881568
rs6587577
rs2282764
rs185174247
rs12451892
rs1985369
rs61748181
rs4530278
rs11584821
rs939916
rs66731853
rs131797
rs11412296
rs117407747
rs6584579
rs6007020
rs10845387
rs28363070
rs79977579
rs117630647
rs10773176
rs41269079
rs611646
rs77231040
rs9398196
rs6536702
rs1003322
rs12412214
rs117512405
rs2977608
rs4498805
rs11769630
rs76666449
rs10905255
rs7705526
rs7209057
rs117034449
rs80324517
rs7790856
rs73730598
rs11699829
rs80116508
rs1957937
rs116863223
rs2538745
rs8102497
rs35640778
rs932002
rs9940099
rs144204502
rs1291143
rs141214782
rs76219171
rs111527438
rs59409453
rs4743037
rs13230646
rs9955360
rs76065543
rs11117354
rs3891167
rs6669563
rs28577594
rs150150565
rs11579626
rs35446936
rs6790988
rs8105767
rs12932179
rs112394943
rs2967355
rs11991877
rs137901416
rs10805346
rs4724
rs34550383
rs12925933
rs17803849
rs143190905
rs17445108
rs77732866
rs202034370
rs11557154
rs9419958
rs6776756
rs45604339
rs145114957
rs3767952
rs871134
rs762679
rs3785074
rs1611236
rs75664430
rs41304832
rs1332941
rs869785
rs11085072
rs2276182
rs55747751
rs2763979
rs17677991
rs61405042
rs13062095
rs28502153
rs182059586
rs111950327
rs4695407
rs6751209
rs1907702
rs5742915
All − MR Egger
All − Inverse variance weighted
−2
−1
0
1
2
MR effect size for
'telomere length || id:ieu−b−4879' on 'IDP_dMRI_TBSS_ICVF_Cingulum_cingulate_gyru
